# Biological–physical interactions are fundamental to understanding and managing coastal dynamics

**DOI:** 10.1098/rsos.230155

**Published:** 2023-07-12

**Authors:** Martin Solan, Tom Spencer, David M. Paterson, Christopher A. Unsworth, Elizabeth K. Christie, Andrew J. Blight, Jenny Brown, Helen Brooks, I. Dougal Lichtman, Xiaoyan Wei, Xiaorong Li, Pete Thorne, Julian Leyland, Jasmin A. Godbold, Charlie Thompson, Megan E. Williams, Andrew Plater, Iris Moller, Laurent O. Amoudry

**Affiliations:** ^1^ School of Ocean and Earth Science, National Oceanography Centre Southampton, University of Southampton, Waterfront Campus, European Way, Southampton SO14 3ZH, UK; ^2^ Cambridge Coastal Research Unit, Department of Geography, University of Cambridge, Downing Place, Cambridge CB2 3EN, UK; ^3^ Scottish Oceans Institute, School of Biology, Sediment Ecology Research Group, University of St Andrews, St Andrews, Fife KY16 8LB, UK; ^4^ Marine Physics and Ocean Climate, National Oceanography Centre, Joseph Proudman Building, 6 Brownlow Street, Liverpool L3 5DA, UK; ^5^ Environment Agency, Tyneside House, Skinnerburn Road, Newcastle Business Park, Newcastle upon Tyne NE4 7AR, UK; ^6^ Department of Geography and Planning, School of Environmental Sciences, University of Liverpool, Liverpool L69 7ZT, UK; ^7^ Energy and Environment Research Group, College of Engineering, Swansea University, Swansea SA2 8PP, UK; ^8^ School of Geography and Environmental Science, University of Southampton, Highfield, Southampton SO17 1BJ, UK; ^9^ Channel Coastal Observatory, National Oceanography Centre, University of Southampton, Waterfront Campus, European Way, Southampton SO14 3ZH, UK; ^10^ Departamento de Ingeniería Hidráulica y Ambiental, Facultad de Ingeniería, Pontificia Universidad Católica de Chile, Santiago, Chile; ^11^ Facultad de Ciencias Biológicas, Pontificia Universidad Católica de Chile, Santiago, Chile; ^12^ Department of Geography, Trinity College Dublin, Museum Building, Dublin 2, Ireland

**Keywords:** science, perspective, coastal protection, nature-based solution, biological–physical interactions, wetland

## Abstract

There is an urgent need to address coastal dynamics as a fundamental interaction between physical and biological processes, particularly when trying to predict future biological–physical linkages under anticipated changes in environmental forcing. More integrated modelling, support for observational networks and the use of management interventions as controlled experimental exercises should now be vigorously pursued.

## 1. The nature of the challenge

Climate change is driving the hazards of accelerating rates of sea-level rise and changing storminess while, at the same time, increased use and settlement of low-lying coasts (less than 10 m above mean sea-level) is raising vulnerability and exposure of coastal habitats, including intertidal sediments, saltmarshes and wetlands. The usual response to coastal flooding risk, pursued over many centuries, has been to build fixed defences such as dikes, sea walls and earthen embankments. More recently, nature-based and eco-engineering solutions have been pursued, such as beach nourishments, sand dune plantings and tidal wetland creation. However, engineered schemes typically incur continual and costly maintenance or replenishment regimes, exacerbated by the need to repeatedly heighten and widen constructions in response to sea-level rise. To retain current levels of coastal risk, modelling suggests that it will be necessary to raise defence heights by 0.5 m by 2050 and by 1 m by 2100 [1]. In some locations, the continued renewal of hard defences remains the only option. Elsewhere, rising costs, and the flood and erosion created by interference with natural coastal dynamics, have raised interest in non-structural responses to coastal change [2]. Yet the paradigm of ‘*Working with natural processes*' raises new challenges. What should be the design rules and implementation practices to successfully work with natural processes in coastal risk management? And, more fundamentally: how well do we understand the sediment dynamic processes—including coupled biological–physical interactions—in the water column, on and within the seabed, and across the water–land interface?

The application of fluid dynamic principles to the entrainment, transport and deposition of coastal sediments is well established, being refined for almost a century following Shields's classic formulation of the 1930s. Similarly, we know a great deal about coastal ecology and the form and function of a range of micro- to macro-organisms within subtidal, intertidal and littoral environments. However, both fields of study have developed in isolation and perspectives are seldom merged. Currently, observations from both approaches are generally piecemeal, biased towards a narrow range of locations (often temperate mid-latitude) and season (often summer), and there is no common standard for obtaining and resolving information. Physical science typically concentrates on monitoring (i.e. one spatial point over time) whereas biological science largely focuses on sampling (one temporal point over space). There is an urgent need to merge these different perspectives and address coastal dynamics as an integrated biological–physical problem. Nature-based coastal protection offers the promise of long-term sustainability as, with adequate sediment supply, coasts have the potential to respond to environmental forcing, including the tracking of rising sea levels. Understanding, and thus aiding, the trajectory of the ecosystem's ability to adapt and maintain functionality is fundamental to long-term maintenance of natural capital and the delivery of coastal ecosystem services. Setting is everything, both from the historical perspective (interventions will be unsuccessful in settings where habitats or ecosystems were clearly not present in the past) and for the future (where there may be opportunities for system migration into favourable new locations). With global environmental change, the behaviour of the natural system will change, needing a flexible approach to nature-based solutions that allow for new options to come into play. There is a need to build a strategy for the long-term observation of ecosystems as they adapt to change that can then be used to validate and build better biological–physical models that, in turn, will allow better prediction into the future. We argue, therefore, that there needs to be a paradigm shift towards the stronger characterization of the spatio-temporal dynamics of coastal systems and that this change in ethos can be of real value to those that live at, work at, or manage the coast. We illustrate our argument with reference to the functioning of temperate saltmarsh, mudflat and subtidal environments.

## 2. Biological–physical interactions: the basis of the challenge

**Figure 1.**
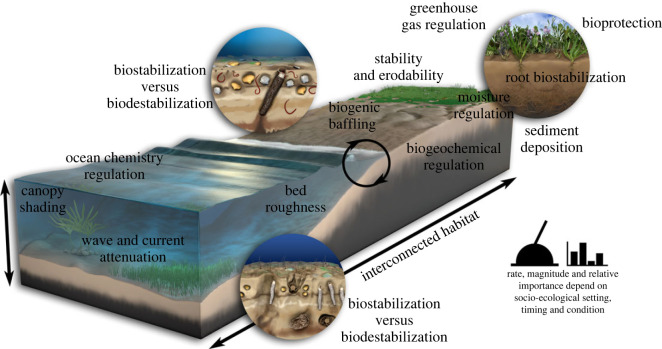
Summary of the salient biological–physical interactions that are fundamental to understanding and managing coastal dynamics, but which are presently poorly constrained as outcomes can be highly dependent on abiotic and biotic context, seasonal timing and/or socio-economic-ecological setting.

At the level of the individual organism, our knowledge of the interactions between biological elements, fluid flows and sedimentation processes are well known (figure 1). Biota ranging from microphytobenthos to macrophytes and sediment-dwelling invertebrates can both biostabilize sediment surfaces or lead to biodestabilization, either passively (e.g. turbulence generated around plant stems) or actively through the bioturbation of surficial sediments. The presence of biota can also lead to the mediation of hydrodynamics through the attenuation of flow, promotion of sediment deposition from suspension through biofilm-enhanced flocculation, increased boundary shear stress and changes to roughness through bio-mediated bedform development. Further, vegetation and biogenic structures modify the benthic boundary layer in a dynamic way, exhibiting both positive and negative feedbacks at a range of spatial and temporal scales [3]. Consequently, bed roughness, a key parameter in the modelling of hydrodynamics and sediment dynamics, is difficult to predict and is an important boundary condition, but is often estimated solely from knowledge of abiotic sediment size distributions or is used as a calibration parameter to match measurements.

## 3. Global change and biological–physical interactions: the future challenge

At the global scale, some consensus is emerging on the broadscale controls on coastal wetland loss, and thus how wetland extent may change in the near future, highlighting the importance of not only the rate of sea-level rise but also sediment supply and accommodation space to survivability. However, within this framework, the likely magnitude of change remains highly contested, ranging from losses of 20–90% versus low losses, and even some gains, in wetland area by 2100 [4]. One difficulty is that global-scale modelling struggles to address the role of the intrinsic feedbacks and complexities described above in modulating external forcing. Thus, for Kirwan *et al*. [5], ‘marsh vulnerability tends to be overstated because assessment methods often fail to consider biological–physical feedback processes'. How will these internal linkages be re-configured in the near future and what redistributions and reorganizations of fauna and sediment/physical habitats will result?

Single-factor climate change metrics, such as global mean temperature increase or global sea-level rise, have little meaning for the explanation of future coastal system scale change. These systemic drivers are subject to spatial variability at the global scale and there are issues of locational downscaling from global to regional, to local, levels. In reality, species responses [6] and biological–physical interactions in coastal settings will be subject to concurrent multiple drivers, both known and currently unanticipated, with the interaction of (i) acute shocks (from changes in tropical and extratropical storminess, associated changes in storm surge frequency and magnitude, marine heat waves, and freshwater flood inputs from future climatologies) and (ii) slow onset, chronic changes, not only from sea-level rise but also including changing rainfall patterns, tides, ocean warming and ocean acidification [7]. These drivers show varying levels of temporal variability which themselves interact with ‘normal’ process levels. Thus, for example, micro-tidal wetlands are thought to be more vulnerable to global change because the sea-level rise signal becomes a larger component of the overall sea-level variability signal, showing an earlier ‘time to emergence’ than in meso- to macro-tidal systems [8].

Considerable challenges lie ahead in hypothecating how species, species assemblages and ecosystems will respond to global change. ‘Step’ models which simply evaluate a future condition against a present condition fail to recognize how futures are arrived at by compounded effects over time. With such cumulative change, different initial model states can rapidly diverge into very different futures. Along any range of pathways, there may be sudden shifts in system state as thresholds are crossed. But it is hard to know what form these thresholds may take, what the ‘distance to threshold’ might be and what happens when thresholds are passed. Finally, the notion of cascades of energy and matter tells us that any kind of climate signal at the coast, and in coastal catchments, is likely to propagate through the landscape over time. The nature and speed of this propagation will be a function of both the degree of connectivity in biological–physical interactions and how this connectivity is organized at the ecosystem scale.

In spite of all these difficulties, some generalizations are possible. A coastal wetland is a three-dimensional near-horizontal platform that occupies an accommodation space within the intertidal zone; thus, any discussion of wetland futures needs to consider both vertical and horizontal change and the possibility of emergent regime shifts (e.g. a shifting balance between vertical keep-up and frontal erosion). In the vertical, biomass productivity affects both the equilibrium marsh elevation and marsh resilience to accelerations in sea-level rise. Latitudinal gradients suggest that warming will increase not only tidal wetland productivity, but also decomposition rates [9]. Mesocosm experiments, observations and modelling [10,11] suggest that, in the short term, warming leads to enhanced carbon storage and vertical accretion. Long-term responses, however, may be more complex as a result of species and/or habitat replacement, as seen in the expansion of tropical mangroves into subtropical salt marshes [12]. For the above-ground canopy, there is evidence that plants are adaptable bio-engineers. Higher shoot flexibility has been demonstrated in response to increasing salinity and inundation stress [13] and increased exposure to hydrodynamic forcing [14], although mechanical resistance associated with enhanced growth under elevated CO_2_ is unlikely to be maintained over the long term [15].

In the lateral cross-shore sense, low, unvegetated mudflats and high, vegetated marshes represent a coupling of two alternative stable states while areas at intermediate elevations are inherently unstable [16]. How will these mudflat–marsh relations evolve with changes in water depth and wave power consequent upon sea-level rise? Modelling for the Venice lagoon suggests that this bipolar system shifts to a permanent non-vegetated state at a rate of sea-level rise of greater than 3.9 mm year^−1^ (for a mixed species marsh) to greater than 5.9 mm year^−1^ (for a marsh dominated by *Spartina* sp.); at greater than 10.6 mm year^−1^, even tidal flats cannot be maintained [17]. Such dynamics will, however, be very location specific as equilibrium elevations will vary with local tidal range, sediment supply, compensatory vegetation growth and wave climate.

Finally, at the present time, wetland dynamics often most strongly reflect the impact of human-induced habitat degradation, fragmentation and restricted landward migration, resulting in reduced adaptive potential to climate-induced change [5]. In estuaries, channel deepening, weir construction, subsidence by ground water extraction and wetland reclamation have altered local bathymetry and significantly changed the hydrodynamics. There have been major implications for shifts in sediment transport/trapping, salinity intrusion, water quality and ecosystem properties [18].

## 4. Solutions and ways forward: meeting the challenge

Interest in the application of nature-based solutions to maintain ecosystem services and mitigate the effects of climate change in support of coastal management challenges is strong. However, the ability to implement such strategies is hampered by the lack of understanding of coupled biological–physical dynamics in littoral, intertidal and subtidal environments [2]. These shortcomings become magnified when trying to predict future biological–physical linkages under new modes of near-future environmental forcing that also require the consideration of concomitant changes in the relative abundance and diversity of functionally important species [19].

It is clear that there is a need to supplant compartmentalism in expertise and promote interdisciplinary solutions, including the use of languages that talk across the environmental disciplines. First, within this broad framework, coastal science needs to better mine existing datasets (e.g. using machine learning), identify and prioritize observations and monitoring to calibrate existing models, and develop new parametrizations of key bio-physical interaction processes. Second, more thoughts could usefully be given to early warning systems, indicators and/or observation networks across key habitats/locations and time scales (including monitoring, which is often curtailed as it is viewed as low priority) to establish the spatio-temporal variations of biological–physical interactions. Third, interventions to reverse the degradation and loss of coastal habitats—such as the managed realignment of low-lying shorelines—should be seen as experimental opportunities in which to learn more about how the biological–physical system works. Thus, for example, modelling of emergent wetland habitats following natural/artificial breaching of coastal defences points to the importance of the interactions between bed elevation (and its control of hydroperiod), hydrodynamics (in terms of both inputs (inlet morphodynamics) and outputs (drainage channel networks)) and sediment erosion/deposition, and also the role of vegetation cover and root depth/structure [20]. As the outcomes of these experiments emerge, it will be important to merge such interdisciplinary information with theory, observation, experiments and replication across systems, so as to establish generality and provide opportunity to minimize the time lag from theory through to evidence-based adoption in practice [21].

The better understanding and more accurate predictions of coupled biological–physical systems have important implications for the maintenance of biodiversity and natural capital and the delivery of ecosystem services, with follow-on benefits and dis-benefits to human livelihoods and well-being. As competing socio-economic and policy demands complicate any decision-making process, we recognize that there is a requirement to probabilistically determine the likelihood and contribution of non-biological–physical variables known to influence coastal dynamics, including anthropogenic feedback loops and trade-offs, to support and feed into the decision-making process. It is well within the potential of the coastal science community—including relevant academia, industry, government and non-government organizations—to deliver these returns if the research framework outlined here can be put in place.

## Data Availability

No datasets were generated or analysed during the current study.
